# Optically transparent and very thin structure against electromagnetic pulse (EMP) using metal mesh and saltwater for shielding windows

**DOI:** 10.1038/s41598-021-80969-3

**Published:** 2021-01-28

**Authors:** Duy Tung Phan, Chang Won Jung

**Affiliations:** Graduate School of Nano IT Design Fusion, Seoul National University of Science and Technology, Seoul, 01811 South Korea

**Keywords:** Engineering, Optics and photonics

## Abstract

An electromagnetic pulse (EMP) with high energy can damage electronic equipment instantly within a wide range of thousands of kilometers. Generally, a metal plate placed inside a thick concrete wall is used against an EMP, but it is not suitable for an EMP shielding window, which requires not only strong shielding effectiveness (SE) but also optical transparency (OT). In this paper, we propose a very thin and optically transparent structure with excellent SE for EMP shielding window application. The proposed structure consists of a saltwater layer held between two glass substrates and two metal mesh layers on the outside of the glass, with a total thickness of less than 1.5 cm. The SE and OT of the structure are above 80 dB and 45%, respectively, which not only meet the requirement of EMP shielding for military purposes but also retain the procedure of good observation. Moreover, the OT of the structure can be significantly improved using only one metal mesh film (MMF) layer, while the SE is still maintained high to satisfy the required SE for home applicants. With the major advantages of low cost, optical transparency, strong SE, and flexible performance, the proposed structure can be considered a good solution for transparent EMP shielding windows.

## Introduction

An electromagnetic pulse (EMP) is a short burst of electromagnetic (EM) wave generated by a nuclear explosion with very high energy that can damage electronic equipment instantly within a wide range. An EMP can destroy electronic equipment, communication systems, radio stations, and radar from a distance of thousands of kilometers from the center of the explosion^1^. The mechanism behind the damage caused by an EMP is that when an EMP arrives at a device, it creates a very strong induced current that enters electronic circuits and destroys them^1,2^. From the point of military defense, an EMP can neutralize and put at risks national and international infrastructure facilities, such as electrical grids and communication networks^3^. Moreover, it can cause severe damage to confidential military intelligence systems by paralyzing military communication equipment^4^. Therefore, developing EMP shielding methods to minimize damage is highly recommended.

Most previous research on the EMP shielding method focused on the SE of concrete structures and multi-waveguides with hexagonal/circular structures because of their excellent shielding performance^5–7^. Concrete structures are the basic frame of EMP shielding facilities to protect shielding rooms from being destroyed by external physical influence. In general, when the concrete structure is constructed, a shielding room is manufactured by metal plates placed on the inside of the concrete structure. However, the use of such a conventional material is not suitable for optical windows to utilize for aeronautic, medical, civilian, and research facilities, which require not only strong SE but also optical transparency (OT). Even if the circular waveguide is optically transparent, its large size leads to a thick structure (at least 15 cm), therefore making it difficult to install on an optical window. Conversely, transparent electrodes (TEs), such as indium tin oxide (ITO)^8^, graphene^9^, carbon nanotubes (CNTs)^10^, metallic nanowires (MNWs)^11^, and metal mesh film (MMF)^8,12^, have also been studied because of their favorable OT and thin-film structure. However, these TEs show a low shielding performance compared with the requirement of EMP applications. Therefore, the development of a transparent EMP shielding structure is imperative, but the existing great challenge is how to balance the inherent contradiction between the high OT and strong SE^8^_._ Specifically, the required EMI SE for EMP shielding is very high at 60 dB for general purposes and 80 dB for military applications^13,14^. Therefore, the design of transparent and effective EMP shielding structures remains challenging.

This research proposes a transparent EMP shielding solution replacing conventional methods using a metal plate or a concrete wall. The proposed method consists of a high-salinity saltwater layer, which is held between two transparent quartz glass layers. To enhance the SE of the structures, one or two metal mesh layers are added to the left and right sides of the quartz glass layers. The proposed method has advantages over its conventional counterparts, including OT, low cost, and ease in fabrication. It is expected to reduce much of the construction costs for EMP-shielded rooms.

## Concept and schematic

**Figure 1:**
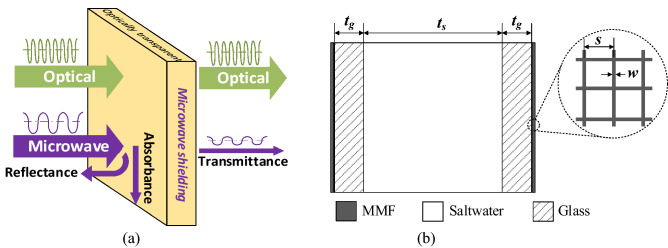
(**a**) Schematic diagram of the EMP shielding window; (**b**) geometry of the MMF/glass/saltwater/glass/MMF structure.

A schematic illustration of the EMP shielding window is shown in Fig. 1a. Most of the EM pulses in radio frequency are reflected and absorbed by the window and rarely pass through it, whereas visible light can transmit without any being affected. To do this task, the window must consist of components that are not only optically transparent but also microwave shielded.

Figure 1b shows the geometry of the MMF/glass/saltwater/glass/MMF structure. As shown in Fig. 1b, the TE consists of a saltwater layer (thickness *t*_*s*_) held between two clear glass quartz layers (thickness *t*_*g*_). To enhance the SE of the structure to meet the requirements of EMP applications, we tape two MMF layers on the outer sides of each glass layer. The mesh width (*w*) and mesh space (*s*) of the MMF are 0.021 mm and 0.234 mm, respectively. Owing to the good transparency of MMF, glass, and saltwater, we expect the OT of the multilayer structure to be retained at high.

## Optical analysis

**Figure 2:**
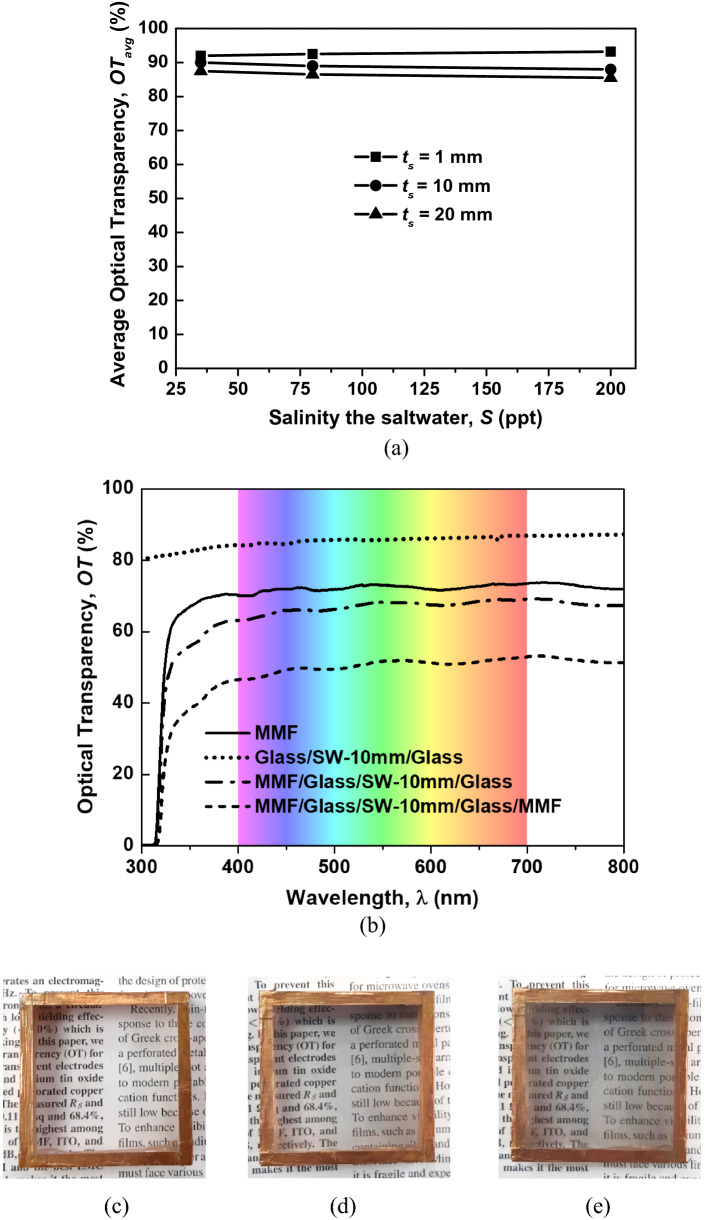
(**a**) Average OT of the glass/saltwater/glass structure with different thicknesses and salinities of the saltwater layer; (**b**) optical transparency spectra of transparent electrodes and structures in the visible range; real pictures of the TEs over texts (*t*_*s*_ = 10 mm): (**c**) glass/saltwater/glass, (**d**) MMF/glass/saltwater/glass, and (**e**) MMF/glass/saltwater/glass/MMF.

Salinity and thickness of saltwater layer are two key parameters that can affect on the OT of the proposed structure. Therefore, first of all we investigate the OT of a glass/saltwater/glass structure with different values of the salinity and thickness. Figure 2a shows the measured OT of glass/saltwater/glass with different salinities and thicknesses of the saltwater layer to study the effect of these parameters on the OT of the structure. The thickness of the saltwater layer varies at 1, 10, and 20 mm, and the salinity varies at 35, 80, and 200 ppt, corresponding to the conductivity of 5, 10, and 20 S/m, respectively. The OT of the structure decreases with the increase in the thickness of the saltwater layer. This can be explained using Beer–Lambert’s law, in which attenuation of the light in saltwater at certain salinity is proportional to the thickness of the saltwater layer.

However, the OT of the structure is nearly unchanged with the increase in salinity. Specifically, when the thickness of the saltwater layer is 1 mm (the absorption can be ignored), the OT of the structure slightly increases. This can be explained by the fact that as salinity increases, the refractive index of saltwater also increases toward the refractive index of the glass (the refractive index of glass is 1.47)^15,16^. Therefore, the refractive index matching between saltwater and glass improves with the increase in salinity, resulting in a decrease in the reflection of light at the interface between them and an increase in transmission through a multilayer structure^17^.

When the saltwater is thin (i.e., 1 mm), the absorption is small and can be ignored. Therefore, the total OT slightly increases with the increase in salinity. However, when the saltwater layer becomes thicker (i.e., 10 and 20 mm), the absorption is significant, which reduces the transmission^18^. This explains why the OT of the multilayer structure is nearly unchanged with the increase in salinity when the thickness is 10 and 20 mm.

Figure 2b shows the measured OT of different electrodes and structures in the visible range. First, we measure the OT of an MMF layer and a glass/saltwater/glass structure for reference. Second, one and two MMF layers are alternately taped to the glass/saltwater/glass structure, and the OT of these multilayer structures are measured. The thickness of the saltwater (*t*_*s*_) and quartz glass layers (*t*_*g*_) is fixed at 10 mm and 2 mm, respectively. The salinity (*S*) of saltwater is 200 ppt.

As shown in Fig. 2b, the OT of MMF is above 68%, with an average value (*OT*_*avg*_) of 70% in the visible range, whereas the glass/saltwater/glass structure shows a very high OT of above 84%, with an average value (*OT*_*avg*_) of 87.5% in the range, which is the highest among the TEs. Moreover, Fig. 2b shows that by adding a single or double layer of MMF to the glass/saltwater/glass, the OT of the multilayer structures significantly decreases. The *OT*_*avg*_ of the MMF/glass/saltwater/glass and MMF/glass/saltwater/glass/MMF structures in the visible range are 64% and 45%, respectively.

Figures 2c–e show real pictures of the three multilayer structures over text. The thickness of the saltwater in the three structures is 10 mm, and the number of MMF layers varies at 0, 1, and 2. The sample without MMF (Fig. 2c) shows a nearly perfect optical performance compared with the realistic one. As shown in Fig. 2d, e, even when the structures with one and two MMF layers show a darker color, they still retain a good enough optical performance for visual observation applications.

## EMI shielding of a multilayered saltwater

### Numerical simulation

In general, the EMI shielding mechanism of a material can be briefly described as the incident EM waves being either reflected or transmitted through the shielding material, but a considerable amount of EM waves may be attenuated because of absorption inside the material. The relevant shielding effectiveness parts for the reflection (*SE*_*R*_), absorption (*SE*_*A*_), and total (*SE*_*T*_) can be expressed as Eq. (1)^19^

1 $$S{E}_{T}=S{E}_{R}+S{E}_{A},$$

where *SE*_*R*_ and *SE*_*A*_ are determined as Eqs. (2) and (3)^19^:

2 $$S{E}_{R}=39.5+10\mathrm{log}\left(\frac{\sigma }{2\pi f\mu }\right),$$

3 $$S{E}_{A}=8.7t\sqrt{\pi f\mu \sigma },$$

where $\sigma$, $\mu$, and $t$ are the electrical conductivity, magnetic permeability, and thickness of the shielding material, respectively. $f$ is the frequency of the incident EM wave.

**Figure 3:**
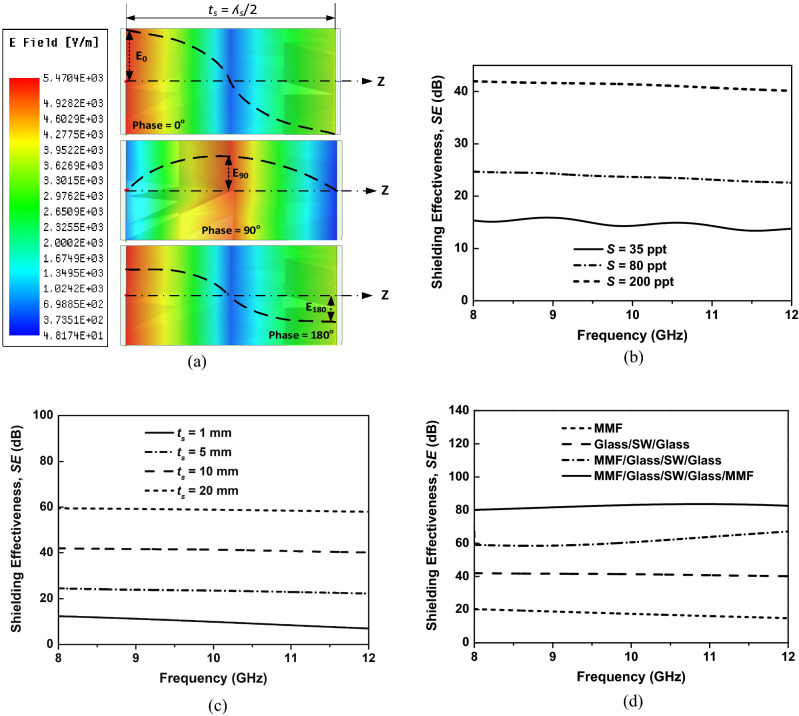
(**a**) E-field distribution of the incident EM wave along with the thickness of the saltwater layer with a thickness of a half wavelength of the EM wave in saltwater (${\lambda }_{s}$); SE of the planar saltwater in the C-band: (**b**) at different salinities (*t*_*s*_ = 10 mm), (**c**) at different thicknesses (S = 200 ppt); (**d**) SE of the four types of TEs in the C-band.

To examine the EM shielding mechanism of the saltwater layer, we extract the magnitude distribution of the E-field of the incident EM wave when it propagates along with the thickness of the saltwater layer. As shown in Fig. 3a, the incident EM wave with a frequency of 8 GHz (@wavelength ${\lambda }_{0}$ of 37.5 mm) propagates along the z-axis from the leftmost to the rightmost of the saltwater layer. Note that when the EM wave propagates in saltwater with a permittivity of 81, the wavelength of the EM wave is *λ*_*s*_ = 4.17 mm. The thickness of the saltwater layer is set to 2.1 mm (*λ*_*s*_/2). Therefore, the E-field distribution is extracted at phases 0°, 90°, and 180°, corresponding to the magnitude at the leftmost, center, and rightmost points (*E*_*0*_, *E*_*90*_, *E*_*180*_), respectively. The magnitude of the incident EM wave significantly decreases when it propagates from the leftmost to the rightmost of the saltwater layer (*E*_*0*_ > *E*_*90*_ > *E*_*180*_). This observation confirms that the absorption contributes crucially to the total EM shielding of the saltwater layer. The EM shielding mechanism of the saltwater layer is different from the metal mesh, in which the reflection mainly contributes to the total EM shielding^8^. A similar phenomenon of EM absorption was reported in the literature^20^.

The shielding effectiveness (SE) of saltwater is a function of its conductivity, which is determined from salinity. Figure 3b shows the simulated SE of the glass/saltwater/glass (*t*_*s*_ = 10 mm) with different salinities. The salinities of saltwater are 35, 80, and 200 ppt, which correspond to the conductivity of 5, 10, and 20 S/m at a room temperature of 25 °C. The SE of the glass/saltwater/glass structure is significantly improved when the salinity increases from 35 to 200 ppt. The increase in the SE of saltwater with the increase in salinity can be explained, as shown in Eqs. (2)–(3), because both reflection and absorption increase with the increase in conductivity of saltwater. Note that the saturated salinity of saltwater at room temperature is 263 ppt^21^. Therefore, we maintain the salinity of the saltwater layer as high as 200 ppt to ensure that the solution cannot be saturated, while the SE is retained at high.

The thickness of the saltwater layer is a basic parameter determining the shielding performance of the glass/saltwater/glass structure. When the thickness of the saltwater layer is less than the skin depth, the shielding performance decreases. In this work, we investigate the effect of the thickness of the saltwater layer on the shielding performance of the glass/saltwater/glass structure, as shown in Fig. 3c. The SE of the glass/saltwater/glass structure is determined using different thicknesses of the saltwater layer while the salinity is fixed at 200 ppt. The SE of the multilayer structure significantly increases with the increase in the thickness of the saltwater layer from 1 to 20 mm. This can be explained by the sheet resistance of the saltwater layer decreasing with the increase in thickness, which contributes to the improvement of the SE. Even the SE can be significantly improved by increasing the thickness of the saltwater layer, as a too-thick structure can limit its application to the optical window. Moreover, the SE of a 20-mm saltwater multilayer is below 60 dB, which is not strong enough for most EMP shielding applications. Therefore, we propose using a 10-mm-thick saltwater layer with MMF to overcome this problem.

Figure 3d shows the SE of glass/saltwater/glass structure with one and two MMF layers. The SE of the MMF and glass/saltwater/glass structure without MMF is also determined for comparison. The saltwater layer shows a good SE above 40 dB in the whole C-band, and it is much better than the MMF, which has an average SE of 19 dB in the band. Although the saltwater layer shows a high SE, it is not strong enough for EMP applications, which require an SE of at least 60 dB. Therefore, we alternately install a single and double layer of MMF on the saltwater layer to improve the SE value. As expected, the SE of the TEs is significantly improved, as shown in Fig. 3d. The MMF/glass/saltwater/glass and MMF/glass/saltwater/glass/MMF show SE values of above 60 dB and 80 dB in the entire interest band, respectively. These values are suitable for EMP applications for commercial and military purposes, respectively.

### Experiment test

**Figure 4:**
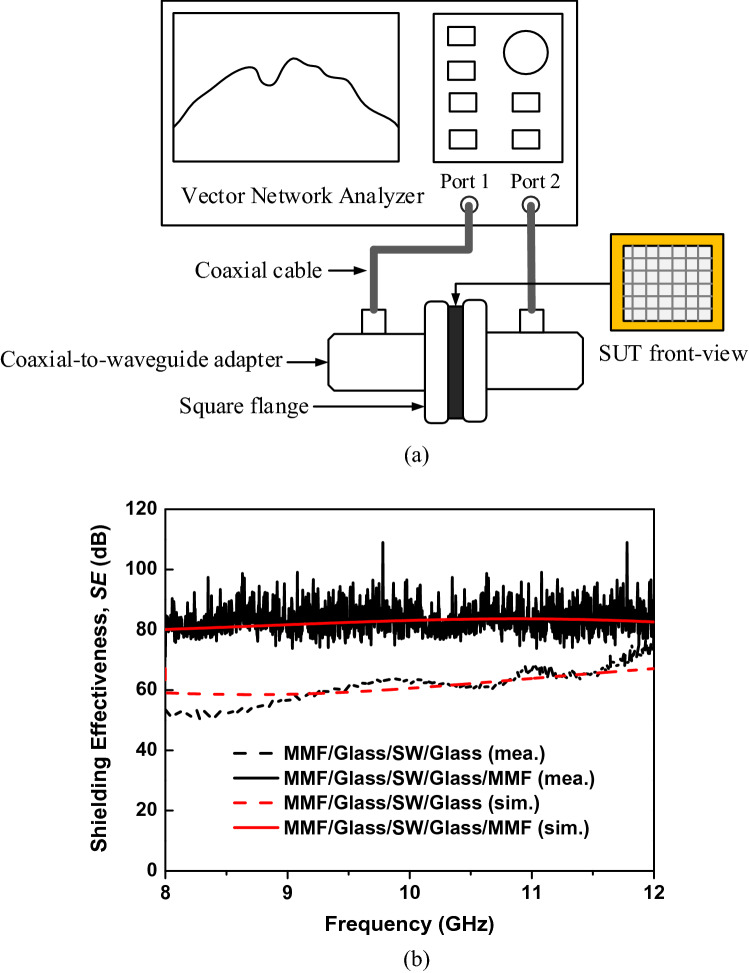
(**a**) Setup for the SE measurement of the structures; (**b**) simulated and measured SE of the saltwater TEs with single and double MMF (*t*_*s*_ = 10 mm).

To verify the simulation results, the measurement of the structure under test (SUT) is conducted using a pair of waveguide-to-coaxial adapters, as shown in Fig. 4a. The SUTs are MMF/glass/saltwater/glass and MMF/glass/saltwater/glass/MMF, with a thickness and salinity of saltwater of 10 mm and 200 ppt, respectively. The saltwater layer is held between two clear quartz glass layers (*t*_*g*_ = 2 mm, $\varepsilon =4.3,\mathrm{tan}\delta =0$). The single and double layers of MMF are alternately installed on the glass layers to fabricate the SUTs, as depicted in Fig. 1b. The MMF layers are very thin (0.005 mm) and can be ignored. Therefore, the total thickness of the multilayered structure is 14 mm. To avoid EM wave leakages between two adapters, we use copper tape to block this gap. Note that the SE can be determined from the power transmission coefficient (*S*_*21*_) in dB as $SE=\left|{S}_{21}\right|$, which is determined using a vector network analyzer connected to the adapters, as shown in Fig. 4a.

Figure 4b shows the measured SE of the TEs, which is in good agreement with the simulation. The measured SE of the structures slightly increases with the increase in frequency. This can be explained by the incident EM wave over 100 kHz: attenuation in saltwater increases with an increase in frequency, leading to a decrease in the wave transmitted through the structures^22,23^. Moreover, the SE of the saltwater layer also slightly increases with an increase in frequency when the thickness of the glass layers (*t*_*g*_) is smaller than a quarter wavelength of the EM wave in the C-band^24^.

The saltwater layer with a double layer of MMF shows a very strong SE over 80 dB, with an average value (*SE*_*avg*_) of 82 dB in the C-band from 8 to 12 GHz. Although the measured SE of the saltwater layer with a single layer of MMF is significantly lower than that of the double-layer MMF, the SE is over 60 dB with *SE*_*avg*_ of 61 dB in the band, which is suitable for EMP shielding for commercial purposes. Table 1 summarizes the average OT and SE of the TEs.

**Table 1:** Summarized OT and SE of the proposed structures.

TEs	*Thickness* (mm)	*OT*_*avg*_ (%)	*SE*_*avg*_ (dB)
MMF	0.005	70	19
Glass/saltwater/glass	14	85	41
MMF/glass/saltwater/glass	14.005	64	61
MMF/glass/saltwater/glass/MMF	14.01	45	82

Table 2 shows the comparison between the proposed structures with the existing transparent electrodes in terms of thickness, working frequency, optical, and SE performance. Most existing TEs are nanowire-based and graphene-based structures that show a very high OT (> 90%). However, the SE of these TEs is low (< 30 dB), which is not suitable for EMP applications. The double metal mesh layer shows a higher SE of 37.61 dB, but it is still not strong enough for EMP applications. On the other hand, the circular multi-waveguide has excellent shielding performance of above 80 dB, but it has a large size, which is a major disadvantage for applications. Compared with the existing transparent TEs and structures, our proposed structure, which is very thin, shows excellent SE that meets the requirement of EMP shielding windows for commercial and military purposes, while the OT performance is retained for the good observation requirement, thus making it the most suitable candidate for EMP shielding window applications.

**Table 2:** Comparison of the proposed structures with other studies.

References	Transparent electrode/structure	Thickness (mm)	Frequency (GHz)	OT (%)	SE (dB)
^7^	Circular multi-waveguide	250	1	N/A	> 80
^24^	Double-layer metal mesh	6.0012	0.15–5	76.35	37.61
^25^	Silver nanowires	N/A	8–12	91.3	28
^26^	Crackle template-based metallic mesh	7	12–18	91	26
^27^	Graphene/metal mesh	N/A	12–18	90	14.1
This work	MMF/glass/saltwater/glass	14.005	8–12	64	61
	MMF/glass/saltwater/glass/MMF	14.01		45	82

## Conclusions

We have successfully fabricated a very thin transparent multilayered structure (less than 1.5 cm) demonstrating excellent EMI shielding performance and sufficient optical transmittance. The major reason for developing a thin and transparent structure is that conventional shielding, such as concrete with a metal plate, is too thick and nontransparent, thus making it inappropriate for applications such as optical windows. The proposed structure consists of a saltwater layer held between two clear glass layers. One or two metal mesh film layers are taped on the outer side of the glasses to enhance the shielding performance of the structure. The OT and SE of the proposed structure are carefully examined by simulation and an experiment, and good agreement is achieved. Specifically, the multilayer structures exhibit a uniform OT of above 44% in the visible band and a strong SE of over 80 dB in the C-band. Moreover, the OT of the structure can be significantly improved by using only one MMF layer, with the SE maintained high to meet the requirement of the EMP shielding for commercial purposes. The SE can be strengthened by increasing the thickness or salinity of the saltwater with a negligible decrease in the OT. With the major advantages of low cost, optical transparency, strong SE, and flexible performance, the multilayered structure using saltwater in glass and MMF on glass can be considered a good solution for transparent EMP shielding applications.

## Methods

### Materials

The ionized salt used in this study was kindly provided by the manufacturer. We prepared the saltwater solution by dissolving the salt in pure water at a room temperature of 25 °C following two steps: (1) pour 100 ml water, which corresponds to 100 g, into a 200 ml glass beaker, and (2) add salt to the beaker and stir. The amount of salt was controlled to achieve the desired salinity using the relationship between them as follows: ${m}_{s}=100S/\left(1000-S\right)$, where ${m}_{s}$ is the amount of salt in grams, and *S* is the salinity of the saltwater solution in parts per thousand (ppt).

### Simulation

**Figure 5:**
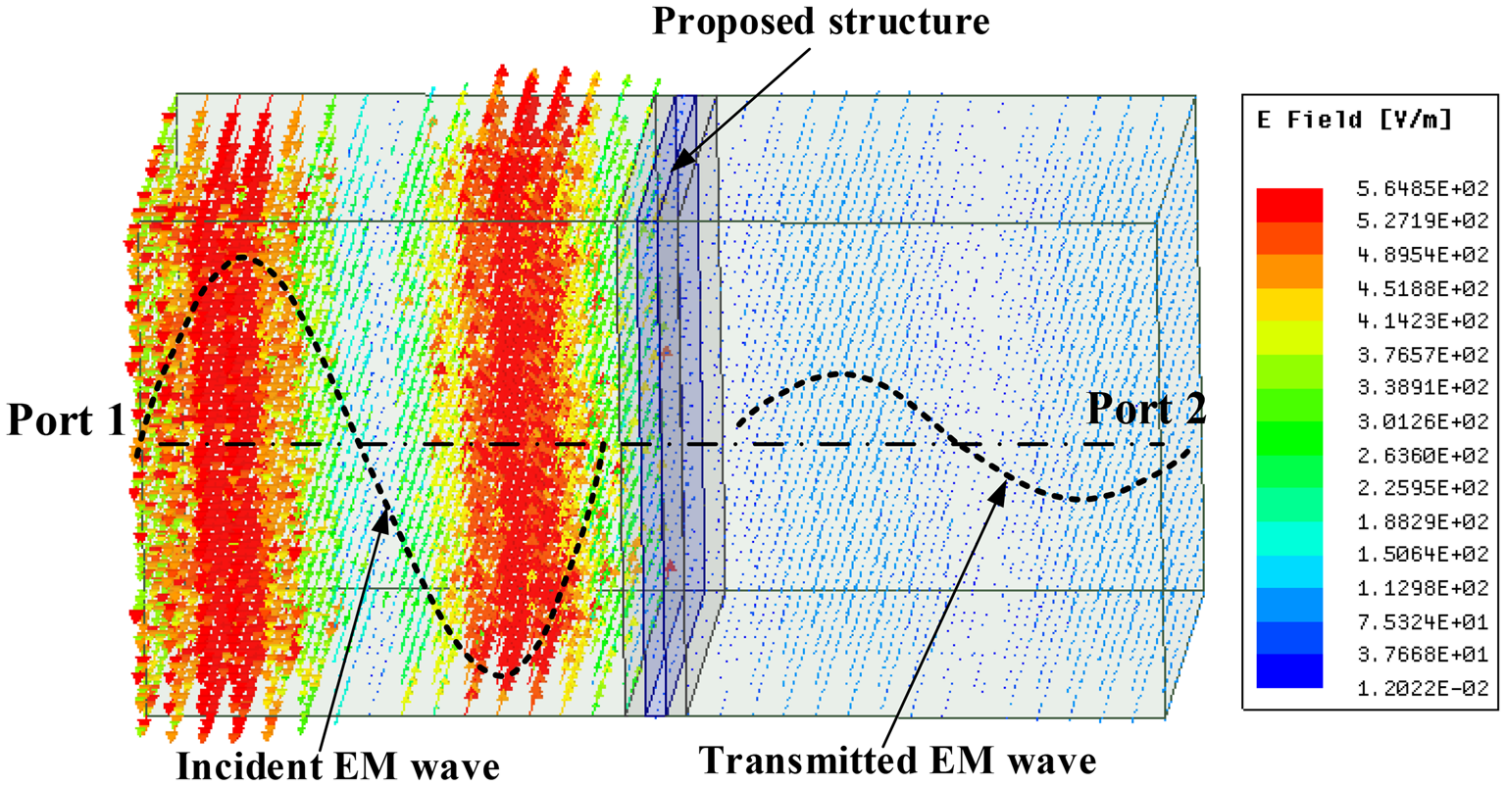
Simulation setup for the SE of proposed structures using Ansys HFSS R2019.1.

The simulation of the EMI SE of the TEs was conducted using Ansys HFSS (high frequency structure simulator) R2019.1. As shown in Fig. 5, the TEs were placed between two wave ports to determine the power transmission (*S*_*21*_) from port 1 to port 2. The simulated SE of the TEs was determined from the *S*_*21*_ (in dB) as $SE=\left|{S}_{21}\right|$.

### Measurement

The OT measurement was conducted using an ultraviolet–visible spectrophotometer (T60 model, PG Instruments Limited Co., UK) connected to a computer. The measurement SE of the TEs in the C-band was measured using a pair of waveguide-to-coaxial adapters (90WCAS) connected to ports 1 and 2 of a vector network analyzer (model E8364C, Keysight Technologies Co., US), as shown in Fig. 4a. The opening cross-section of the adapters was 22.5 mm × 10 mm, corresponding to a bandwidth of 8–12 GHz. The SUT had the same dimension fit to the outer size (cover flange) of the adapters (45 mm × 45 mm). The SUT was fabricated using 2-mm-thick quartz glass ($\varepsilon =4.3,\mathrm{tan}\delta =0$) as the substrate and a 10-mm-thick saltwater layer ($S=200 ppt @\sigma =20 \mathrm{S}/\mathrm{m}$) as the conductive layer. The total thickness of the SUT was 14 mm. Therefore, to avoid leakage waves, copper tape was used to block this gap. The measurement procedure of the SUT includes two steps: (1) connect the two adapters directly and measure the transmission coefficient *S*_*210*_, and (2) separate the two adapters using the SUT and measure transmission coefficient *S*_*21S*_. The measured SE of the SUT was determined from the measured transmission coefficients as $SE=\left|{S}_{21S}\right|-\left|{S}_{210}\right|$.
